# Antimicrobial resistance control activities at a tertiary hospital in a low-resource setting: an example of Queen Elizabeth Central Hospital in Malawi

**DOI:** 10.3389/frabi.2023.1202256

**Published:** 2023-09-20

**Authors:** Patrick Kamalo, Pui-Ying Iroh Tam, Thokozani Noniwa, Chikumbutso Mpanga, Chanizya Mulambia, Ethwako Phiri, Dingase Kumwenda, Ed Phillipo, Samantha Lissauer, David Kulapani, Christina Mwinjiwa

**Affiliations:** 1 Department of Neurosurgery, Queen Elizabeth Central Hospital, Blantyre, Malawi; 2 Department of Paediatrics and Child Health, Queen Elizabeth Central Hospital, Blantyre, Malawi; 3 Department of Paediatrics and Child Health, Kamuzu University of Health Sciences, Blantyre, Malawi; 4 Paediatrics and Child Health Research Group, Malawi-Liverpool Wellcome Programme, Blantyre, Malawi; 5 Division of Clinical Sciences, Liverpool School of Tropical Medicine, Liverpool, United Kingdom; 6 Department of Laboratory Medicine, Queen Elizabeth Central Hospital, Blantyre, Malawi; 7 Department of Trauma and Orthopaedic Surgery, Queen Elizabeth Central Hospital, Blantyre, Malawi; 8 Department of Internal Medicine, Queen Elizabeth Central Hospital, Blantyre, Malawi; 9 Department of Obstetrics and Gynaecology, Queen Elizabeth Central Hospital, Blantyre, Malawi; 10 Dept of Infection and Immunity, University of Liverpool, Liverpool, United Kingdom; 11 Department of Pharmacy, Queen Elizabeth Central Hospital, Blantyre, Malawi

**Keywords:** antimicrobial resistance, antimicrobial stewardship, infection prevention and control, low-resource settings, low- and middle-income countries, leadership, AMR control

## Abstract

**Background:**

Addressing AMR has been most problematic in low- and middle-income countries, which lack infrastructure, diagnostic capacity, and robust data management systems, among other factors. The implementation of locally-led efforts in a low-income country to develop sustainability and build capacity for AMR control within the existing infrastructure has not been well documented.

**Methods:**

We detail current AMR control initiatives at Queen Elizabeth Central Hospital, a tertiary referral government hospital in Malawi with limited resources, and present the activities accomplished to date, lessons learned, and challenges ahead.

**Results:**

The key areas of AMR control initiatives that the group focused on included laboratory diagnostics and surveillance, antimicrobial stewardship, infection prevention and control, pharmacy, leadership, education, and funding.

**Discussion:**

The hospital AMR Control Working Group increased awareness, built capacity, and implemented activities around AMR control throughout the hospital, in spite of the resource limitations in this setting. Our results are based on the substantial leadership provided by the working group and committed stakeholders who have taken ownership of this process.

**Conclusion:**

Limited resources pose a challenge to the implementation of AMR control activities in low- and middle-income countries. Leadership is central to implementation. Future efforts will need to transition the initiative from an almost fully personal commitment to one with wider engagement to ensure sustainability.

## Background

Antimicrobial resistance (AMR) has reached alarming levels globally, and is estimated to cause 4.95 million deaths globally, with the highest rates being in sub-Saharan Africa (Antimicrobial Resistance C, 2022). Addressing AMR has been most problematic in low resource settings, which lack infrastructure, diagnostic capacity, and robust data management systems, among other factors.

As a government hospital based in a low income country (LIC) setting, Queen Elizabeth Central Hospital (QECH) has played a central role in documenting the emergence of AMR in Malawi. QECH, established in 1964 and the largest tertiary care hospital in Malawi, serves a population of over 7 million people, handles over 400,000 outpatients in its various specialized clinics and admits over 75,000 patients annually. Health services are offered free of charge to all patients, and the government with support from various donors funds all services through the Ministry of Health and the Ministry of Local Government. The QECH hospital laboratory was set up in 1954, providing microscopy services initially with eventual scale-up to full culture and susceptibility testing. In the 1990s, the Malawi-Liverpool Wellcome Programme (MLW), a research institution based at QECH, started offering partial microbiology services support in form of high-quality and robust blood and cerebrospinal fluid (CSF) surveillance to the medical and paediatric wards at QECH. Through this surveillance programme, MLW documented a concerning increase in AMR at QECH from 1998-2016 (Musicha et al., 2017), with an increase in resistance to first-line antimicrobials to over 90% for some Gram-negative pathogens in children (Iroh Tam et al., 2019). More recently, carbapenemase-producing *Enterobacteriaceae* have been detected at QECH (Lewis et al., 2020), possibly attributed to the increasing use of carbapenems in tertiary hospitals, after these were included on the Ministry of Health essential medicine list in 2015.

At about the same time, doctors practicing in other departments which are not covered by MLW microbiology services noticed an increasing incidence of infections that were resistant to commonly available antibiotics in the hospital. In response to this, in January 2019, a one-day workshop was held at QECH where a commitment was made to combat AMR at QECH. The workshop also resolved to create an AMR Control Working Group, among other interventions, with the aim of minimizing the emergence of AMR, developing a hospital laboratory microbiological surveillance platform beyond the blood and CSF culture surveillance provided by MLW, and increasing awareness and visibility of AMR initiatives and management.

AMR control, antimicrobial stewardship (AMS), and infection prevention and control (IPC) initiatives have been described extensively in high-income country settings (Zingg et al., 2019). However, the implementation of locally-led efforts in an LIC to develop sustainability and build capacity for AMR control within the existing infrastructure, which this working group represents, has not been well documented. We detail current AMR control initiatives at a tertiary referral government hospital in Malawi with limited resources, and present the activities accomplished to date, lessons learned, and challenges ahead (Table 1). We discuss each of these briefly in turn.

## Laboratory diagnostics and surveillance

As a tertiary referral centre for the southern region of the country, QECH requires laboratory diagnostics to serve its patient population, and good quality, robust, and representative microbiology data are needed to develop locally relevant AMS and IPC interventions. The collapse of funding for health care delivery in Malawi in the 1990s (Kalipeni, 2004) resulted in the inability of the hospital to procure reagents for culture and susceptibility testing, and the dwindling of microbiology services at QECH laboratory to rudimentary services where only microscopy could be performed. What emerged from that period is microbiology support that has been provided by MLW, a research institution adjacent to QECH with an ISO-accredited laboratory. Currently, the MLW laboratory processes 1,700 blood and 400 CSF cultures from QECH patients in the medical and paediatric wards every month, providing surveillance and patient care at no cost to the hospital.

MLW surveillance does not include other sections of the hospital (surgery, obstetrics and gynaecology) nor body fluids other than blood and CSF. To address this gap, the QECH microbiology laboratory has been developing the capacity to process wound swabs, pus, urine, and other body fluid cultures from all patients that come to QECH. However, comprehensive isolate and susceptibility testing has usually remained a challenge due to a lack of equipment, susceptibility tests, and reagents.

In 2018, Malawi received a grant from the Fleming Fund which was aimed at supporting microbiology services in low- and middle-income countries (LMICs). QECH, together with all public tertiary hospitals in Malawi, was included as one of the beneficiaries of this support. This support has included the rehabilitation of the hospital microbiology laboratory section, establishing provision of equipment performing blood culture tests, training in accurate isolate identification, susceptibility testing according to currently available antibiotics, and also surveillance for AMR trends. The Fleming Fund also provided start-up reagents for 3 months, and after that, the hospital took over those costs. The support did not include the hiring of new staff or extra money for the existing microbiology laboratory staff.

As QECH re-establishes a robust and quality-assured microbiology service, there are a number of challenges that need to be addressed, including staff shortages, patient specimens disappearing between patient collection to delivery to the laboratory, regular stockouts of reagents, and quality control of microbiological testing. To address the latter issue, the laboratory has been participating in different external quality assessment schemes, such as the National Health Laboratory Service (NHLS), CDC, and External Quality Assessment for Africa (EQuAFRICA), and has been earmarked for Southern Africa Development Community Accreditation Services (SADCAS) and is expected to have its initial assessment towards the end of 2023.

## Antimicrobial stewardship

Targeted AMR efforts have become even more urgent in the post-COVID-19 era, which has been associated with increased antibiotic use (Rawson et al., 2020; Karami et al., 2021). AMS programmes have been shown to reduce costs, decrease hospital length of stay, and reduce the burden of AMR (Nathwani et al., 2019; Mahmoudi et al., 2020; Gebretekle et al., 2021). However, this impact has been demonstrated primarily in high-income countries, that have the resources to conduct intensive surveillance. In LMICs, scarce resources impede the development of a robust surveillance platform that is needed to provide a critical baseline to measure the effect of interventions. One method for measuring antimicrobial usage that is useful, practical, and economical is the point-prevalence survey (PPS) (Gharbi et al., 2016), which has been successfully used in LMICs to provide actionable data (Singh et al., 2019). The AMR Control Working Group at QECH has conducted several PPS to monitor antimicrobial usage across the hospital and used that to inform prescribing practices.

Overuse of broad-spectrum antibiotics has been documented in other LMIC hospitals (Sonda et al., 2019), and the development of rational antimicrobial guidelines to guide clinicians in an era of increasing AMR is urgently needed. Prior AMS interventions at QECH have been demonstrated to be suitable and sustained (Lester et al., 2020), but this has only been limited to a few wards in the hospital. One of the goals of the AMR Control Working Group is to address hospital-wide AMR issues, including the development and adherence to AMS guidelines, as would be relevant to a teaching hospital. Achieving this will require the participation of a multidisciplinary team including infectious diseases physicians, clinical microbiologists, pharmacists, subspecialty physicians, and epidemiologists, who recognize that AMR is a public health emergency, and are committed to sustained partnerships and evidence-based practice. More importantly, representative and reliable microbiology data are required from all sections of the hospital, in order to develop a comprehensive hospital antibiogram. Currently, data from the adult surgical wards, and the obstetrics and gynaecology wards are inadequate.

In addition, AMS rounds on patient wards provide an opportunity for clinicians to interact with the AMS team, ask questions on optimal antimicrobial usage and address patient management issues. AMS rounds in paediatric hospital care were found to encourage participants to critically evaluate antimicrobial choices and to engage in discussion with the AMS team, and also for participants to gain confidence in prescribing antimicrobials (McCreary et al., 2021). At QECH, paediatric AMS rounds have been formally conducted since January 2022, and include the participation of a paediatric infectious diseases specialist and clinical microbiologist. These weekly paediatric rounds target the sickest patients in the intensive care, surgical and medical high dependency, and malnutrition units. The service has been well received by the clinical teams and resulted in a change of management in most cases. There is a need for similar interventions in adult specialties, but microbiologists and infectious disease specialists are a scarce resource in this setting (Chetty et al., 2022).

## Infection prevention

Particularly for vulnerable populations such as neonates, hospital-acquired infections with AMR pathogens contribute to an extended hospital stay, increasing hospital costs, and are a leading cause of morbidity and mortality (Dramowski et al., 2022). However, while there is strong evidence for IPC and its impact on infections, there is a limited evidence base for the effectiveness of IPC interventions in LMIC settings (Fitzgerald et al., 2022). There are ongoing IPC interventions on the QECH neonatal unit, led by the clinical teams and supported by an ongoing research study. QECH has an IPC committee and specific units such as the paediatric surgical intensive care unit have set up their own IPC committees to address ward-specific issues. However, protected time and available resources to support general IPC initiatives are lacking.

## Pharmacy

Engagement of the hospital pharmacy including an understanding of antibiotic availability and consumption is essential in developing robust antimicrobial prescribing practices in the context of high AMR rates in hospital settings. Some (small) hospitals have demonstrated that, in settings where there are no infectious diseases physicians or microbiologists, pharmacist-led AMS programmes can be effective in reducing the consumption of antimicrobials and therefore retard the development of AMR (Cantudo-Cuenca et al., 2022).

At QECH, the hospital pharmacy is supported by the Ministry of Health and deals with roughly 10,000 prescriptions per month. More than half of these prescriptions contain an antibiotic. Medicines are provided free of charge to patients and costs are borne by the Ministry of Health.

Medicines are managed by qualified pharmacists and pharmacy technicians, who provide patient education and counselling on proper use of antimicrobials. In addition to coordinating the QECH Drug and Therapeutic Committee, a hospital committee involved in the selection, quantification and procurement of medicines and antimicrobials, the pharmacy has been promoting and encouraging the use of prescription pads for all antimicrobial prescriptions, and the exchange of empty vials and use of patient files when collecting antimicrobials from the pharmacy.

Challenges include inadequate staff, the limited spectrum of antimicrobials on the central hospital essential medicines list provided by the Malawi Ministry of Health, and frequent stockouts of medicines on the essential medicines list. This makes it challenging to implement the World Health Organisation AWaRe principles which encourage the widening of the spectrum of antibiotics available for use, in order to reduce the overuse of popular antibiotics and impact on AMR (WHO, 2021).

## Education

Currently, medical trainees receive about 60 hours of education in pharmacy as medical students, but only a handful of lectures on AMS. Clinical officers, who are part of a 3-year education programme, receive even less. Therefore, clinician antimicrobial prescribing practices tend to be based on hospital guidelines and observed practice, whether correct or not. Focused AMS training may need to be considered for specific units such as pharmacy, and wards such as Accident & Emergency, where the majority of initial antibiotic regimens are prescribed. It is important to note that in the training for the older graduates, whom are now supervisors and mentors of the new medical graduates, AMS was not covered in their curriculum. This means that senior staff need to be educated and trained first on these principles before they can influence the prescribing practices of the younger generation.

## Leadership

Leadership and good governance are needed for sustainability, as this ensures retention of skilled staff, maintenance of equipment, and adoption of a quality mindset (Wertheim et al., 2021). AMR initiatives in LMICs that have been sustained and expanded after the project ended have depended on strong leadership (Malania et al., 2021). In addition, strong governance is tied to accountability, transparency, sustainability, engagement, equity, and international collaboration. In the case of the QECH AMR Control Working Group, a consultant neurosurgeon is the champion and lead and performs his working group duties without any protected time. Four local specialists from paediatrics, surgery, obstetrics and gynaecology, and internal medicine were selected to be trained as AMR officers, with duties to champion AMR control activities in their respective departments. Two hospital-wide workshops were held, one in 2019 and another in 2022, to raise awareness on the issue of AMR at QECH, plan and get input on AMR activities and initiatives, review results from the hospital PPS and surveys, and discuss the progress, challenges, and status of the hospital laboratory, pharmacy, and related departments.

## Funding considerations

In resource-limited settings such as in Malawi, investment in human resources, training, surveillance, monitoring, quality control, and leadership are acutely needed, in order to build capacity, and incentivize and empower local personnel. While much of the AMR control activities have been a local initiative, external funding from the Fleming Fund and Pfizer has supported some AMR activities, including the time of AMR champions and hosting of hospital-wide AMR control awareness activities. However, internal funding is scarce and hospital management and leadership will need to prioritise funds to the QECH AMR Control Working Group to ensure sustainability.

In summary, the key areas of AMR control initiatives that the group focused on included laboratory diagnostics and surveillance, AMS, IPC, pharmacy, leadership, education, and funding. Since its inception, the QECH AMR Control Working Group has made considerable headway in increasing awareness, building capacity, and implementing hospital activities around AMR control, in spite of the resource limitations in this setting. Our status is similar to AMS programmes in public sector hospitals in KwaZulu Natal, South Africa, where only 75% of hospitals have an AMS committee, 47% have a formal written statement of support from the leadership, and 7% receive budgeted financial support (Chetty et al., 2022). What has been central to the progress at QECH is the substantial leadership provided by the working group and its committed individuals who have taken ownership of this process. To ensure sustainability, future efforts will need to transition the initiative from one being an almost fully personal commitment to being meaningfully supported by the hospital through the provision of protected time for AMR activities but also with local funding for AMS activities.

## Figures and Tables

**Table 1:** Challenges, activities accomplished, and lessons learned with AMR control activities at Queen Elizabeth Central Hospital in Malawi.

Area	Issue	Challenges	Activities accomplished	Next steps and/or lessons learned
Laboratory diagnostics and surveillance	Patient specimens going missing	Clinicians are not aware of missing samples until they check the system a few days later, at which point repeat sampling may not be feasible.	Creation of a laboratory users social media group where alerts on microbiology laboratory activities are posted. It remains a challenge of time efficiency, reducing risk of human error, and deciding which and how many people should be included on the group.	Identify gaps in specimen transfer from ward to lab, and place steps for improved monitoring and accountability, including a process to check results robustly, and the development of a hospital electronic medical records system.
	Hospital laboratory running out of reagents	Unavailability of reagents at the central medical stores. Procurement challenges.		Advocate for central procurement of reagents as part of the national AMR control programme.
	Hospital laboratory antimicrobial susceptibility profiles discordant with MLW	QECH hospital laboratory data		Establish quality control between the two laboratories
Antimicrobial stewardship	Baseline date of antimicrobial prescribing practices	Set up point prevalence survey (PPS).	2 hospital-wide PPS done in one year. Selected staff trained in conduct of PPS, and also for support of other AMR activities.	Continued monitoring with serial PPS.
	Baseline knowledge of health workers of AMR control issues	Set up survey to assess knowledge and reported practices of health care workers on AMR control.	3 surveys conducted to date.	Continued monitoring with serial surveys.
	Case file audit to monitor quality of clinical notes ad recording by clinicians	Set up audits of patient case files to monitor the quality of documentation and AMR control best practices by clinicians.	3 audits completed to date.	Continued monitoring with serial audits.
		Poor completion of medical charts with missing data. Case management forms with 17 variables were listed on the order form for culture and susceptibility testing. However, forms were incompletely filled in by the ordering clinicians.	Training of staff organised and conducted but there was poor attendance (~50%) by clinical staff	Sensitisation of not just heads of departments but clinicians, with presence at dept meetings, serial awareness talks, presence, and engagement on the wards, constant education and feedback. Devise other innovative ways of dissemination of AMR control messages, like social media and inter-departmental collaborations.
	Regular feedback with culture data	Set up notification system of positive culture data requires linkages with microbiology lab and data management services.	MLW sends weekly notifications of positive blood cultures in paediatrics. QECH main laboratory sending real time notifications through a WhatsApp group.	Need for timely access to all results, negative as well as positive. Concern that more frequent notifications will lead to notification fatigue. Linking of clinical data (Blantyre Institute for Neurological Sciences (BINS) electronic medical records system) and QECH laboratory data (LIMS) electronically.
	Antimicrobial stewardship rounds	Overuse of broad-spectrum antibiotics.		Development of AMS guidelines, revised and updated to reflect current AMR profiles.
		Continuation of antibiotics in the setting of negative cultures.		Development of AMS guidelines, revised and updated to reflect current AMR profiles
		A&E is site of majority of initial antimicrobial prescriptions.		Development of AMS guidelines to reduce unnecessary antimicrobial prescriptions.
		Comprehensive hospital-wide AMS activities	AMS rounds implemented on paediatric wards only.	Identify clinicians and/or trained staff (pharmacists) to lead AMS rounds on other wards.
Infection prevention	IPC interventions	Complex interventions engaging multiple cadres of health care workers and staff behavioural change.	IPC interventions developed in neonatal unit through multi-step process led by government staff	Implement intervention alongside training package and monitor fidelity.
		No clear guidance on important interventions in LMIC settings.		
		Require consistent resource supply, e.g. soap, chlorine, buckets and availability of single use items (e.g. nasal prongs)		IPC prioritization within hospital settings to allocate scarce resource.
Pharmacy	Stockout of antibiotics	Inconsistent availability of antibiotics at the central medical stores.		
	Limited selection of antibiotics at the pharmacy resulting in overuse of the few available antibiotics, overuse of broad-spectrum antibiotics, and poor adherence to available guidance.	Limited availability of alternative antibiotics		Lobby government to widen the rage of antibiotics in relevant hospitals to reduce pressure on the commonly used antibiotics.
Education	Training and education of clinicians	Clinicians with limited antimicrobial prescribing knowledge.		Development of AMS guidelines, and training of clinicians on AMS guidelines. Widen the selection of available antibiotics, and provide more training on appropriate and judicious use.
Leadership	AMR leadership	Leaders are not given protected time to initiate, supervise, and support AMR initiatives	Appointed AMR officers Support from external funders for AMR officers to commit 20% of their time to AMR activities, and were trained on hospital AMR guidelines and protocols, conduct of surveys on health care awareness of AMR issues, patient file reviews to assess quality of documentation of AMR, education campaigns on AMR control best practices.	Recruit more AMR officers into the programme. Include other departments (pharmacy, laboratory, A&E, infection prevention) in AMR control agenda.
	Involvement and visibility of management	Initial communications on case management of culture-positive patients were sent by email to the HODs, but later follow-up suggested that awareness was low.	Face-to-face meetings, regular presence at dept meetings,	Introduce AMR control in dept morbidity and mortality meetings.
Funding	Hospital budget	Limited budget to appoint staff focused on AMR activities. Limited budget to support additional initiatives, therefore working group has depended on outside funding	Support from external funders to host hospital-wide AMR control awareness activities.	Lobby hospital management and leadership to allocate funds for AMR control awareness activities.

## Data Availability

The original contributions presented in the study are included in the article/supplementary material. Further inquiries can be directed to the corresponding author.
